# Eating, Sleep, and Social Patterns as Independent Predictors of Clinical, Metabolic, and Biochemical Behaviors Among Elite Male Athletes: The EROS-PREDICTORS Study

**DOI:** 10.3389/fendo.2020.00414

**Published:** 2020-06-26

**Authors:** Flavio A. Cadegiani, Claudio E. Kater

**Affiliations:** Adrenal and Hypertension Unit, Division of Endocrinology and Metabolism, Department of Medicine, Federal University of São Paulo Medical School, São Paulo, Brazil

**Keywords:** hormonal conditioning, endocrinology of physical activity, sports endocrinology, hormones and sports, Endocrine and Metabolic Responses on Overtraining Syndrome (EROS) study, overtraining syndrome

## Abstract

**Objectives:** Physiological hormonal adaptions in athletes and pathological changes that occur in overtraining syndrome among athletes are unclear. The Endocrine and Metabolic Responses on Overtraining Syndrome (EROS) study evaluated 117 markers and unveiled novel hormonal and metabolic beneficial adaptive processes in athletes. The objective of the present study was to uncover which modifiable factors predict the behaviors of clinical and biochemical parameters and to understand their mechanisms and outcomes using the parameters evaluated in the EROS study.

**Methods:** We used multivariate linear regression with 39 participants to analyze five independent variables—the modifiable parameters (caloric, carbohydrate, and protein intake, and sleep quality and duration of concurrent cognitive activity) on 37 dependent variables—that were elected among the parameters evaluated in the EROS study.

**Results:** Carbohydrate intake predicted quick hormonal responses to stress and improved explosive responses during exercise. Protein intake predicted improved body composition and metabolism and caloric intake, regardless of the proportion of macronutrients, predicted muscle recovery, and alertness in the morning. Sleep quality predicted improved mood and excessive concurrent cognitive effort in athletes under intense training predicted impaired metabolism and libido.

**Conclusions:** The results support the premise that eating, sleep, and social patterns modulate metabolic and hormonal function, clinical behaviors, and performance status of male athletes, and should be monitored continuously and actively to avoid dysfunctions.

## Introduction

Physical activity has multiple benefits, including decreased risk for multiple diseases, increased life expectancy, and improved quality of life (1–3). To achieve these benefits, a balance among major lifestyle habits, including training, resting, and eating patterns, is critical. Classically, healthy habits include sufficient caloric, protein, and carbohydrate intake, adequate sleep quality and duration, and avoidance of concurrent excessive psychological or cognitive stress, especially during moderate-to-intense training (4). However, our understanding of whether and how these habits may predict and modulate behaviors of hormonal, metabolic, clinical, and other biochemical parameters is poor. Conversely, it has been extensively reported that excessive training may disrupt physiological processes, induce multiple dysfunctions, and eventually lead to overtraining syndrome (OTS). It is also uncertain whether and how eating, social, and sleep patterns disrupt adaptive physiological changes in athletes, leading to the pathophysiology of OTS (4, 5).

Unlike the cardiovascular and musculoskeletal systems, extensively described in athletes, the peculiarities and not fully elucidated hormonal and metabolic adaptations to sports challenged the research on the endocrinology of physical activity and sport. The hormonal adaptations to physical activity were poorly understood, and consequently, research of biochemical markers on OTS has been compromised since levels expected for athletes were unknown.

Therefore, we conducted the Endocrine and Metabolic Responses to Overtraining Syndrome (EROS) studies (6–9), in which we evaluated 117 parameters, including exercise-independent hormonal responses to stimulation tests, basal hormones, muscular, immunologic, classic inflammatory, lipid, and hematologic parameters, body composition and metabolic rates, psychological, sleeping, and detailed eating patterns, in both athletes affected by OTS and healthy athletes, comparing to healthy athletes and healthy sedentary, respectively, in a three-arm study. The EROS study was designed to address some of the challenges and limitations of the assessment methods of the studies on athletes and unveil novel insights from overcoming the methodological limitations, including: (1) The employment of two control-groups, of healthy athletes and also of healthy sedentary, which allowed the analysis of the results from a more comprehensive perspective, since the simultaneous evaluation of the influence of the physical activity under healthy state and how this influence is altered under OTS is possible due to the concurrent comparisons with sedentary controls. In addition, findings on healthy athletes, when compared to non-active participants, were also relevant, particularly for the present study; (2) In the case of the OTS group, recruitment of athletes suspected of OTS in real life, aiming to evaluate actual and natural-occurring OTS, strictly diagnosed with diagnostic flowchart proposed by the latest guideline on OTS, including the exclusion of confounding diagnoses and the *sine-quo-non* presence of the key criteria of a minimum of 10% reduction in sports-specific performance; (3) Exclusive employment of extensively validated and standardized tests, and endorsed by specialized societies, in order to have reliable results and conclusions; (4) Performance of exercise-independent stimulation tests, aiming to avoid sub-optimized responses due to differences in performance, which also allow comparisons with non-physically active controls; (5) Concurrent evaluation of multiple and broad different aspects for the identification of which sorts of dysfunctions are present in OTS, in order to allow further analyses of how these dysfunctions correlate and interact in both development of OTS and in normal physiology, detection of independent triggers of OTS, and possible determinants of behaviors between the parameters evaluated in all athletes.

In the EROS study, we analyzed three groups: healthy athletes, OTS-affected athletes, and non-athletes. The main objective of this EROS study was to understand the behaviors associated with multiple parameters in male elite athletes, and how these parameters are modified by the presence of OTS by comparing the OTS-affected and healthy athletes with the sex-, age-, and body mass index (BMI)-matched non-athletes.

Each parameter was compared among the three groups, for which both overall and pairwise comparisons were conducted, aiming to understand the behavior of each evaluated marker in healthy athletes by comparing them with the non-athletes, and OTS athletes, thereby comparing affected with healthy athletes.

The changes in the methodology of the EROS study allowed the identification of novel findings and the clarification of previously inconsistent results. The most remarkable findings unveiled by the primary arms of the study, which included the EROS-HPA axis (6), the EROS-STRESS (7), the EROS-PROFILE (8), the EROS-BASAL (9), as well as the novel insights in OTS (10), the findings in high intensity functional training (EROS-HIFT) (11), and the demonstration of enhancement of hormonal responses to stimulations (12), include:

1. Through a 7-day thorough and precise diet record, athletes affected by OTS had a prior diet of ~2 times less carbohydrates, two times less protein, and two times less overall caloric intake shown as g/kg/day, g/kg/day, and kcal/kg/day, respectively, when compared to healthy athletes, and three times less carbohydrate than sedentary controls;
2. Healthy athletes had better sleep quality (but not longer) and had shorter working or studying duration (h/day);
3. At an insulin tolerance test (ITT), performed to evaluate hormonal responses to a stressful stimulation (hypoglycemia), healthy athletes disclosed optimized and prolonged GH and cortisol responses compared to non-physically active controls, and was the only group to disclose a significant response of prolactin to stimulations, which was lost under OTS;
4. Direct stimulation of the adrenal glands using a synthetic ACTH did not yield any difference between healthy and affected athletes, and sedentary;
5. Testosterone levels were higher in healthy athletes than both sedentary and OTS-affected athletes;
6. The testosterone-to-estradiol ratio, an indirect marker of aromatase activity, was ~2 times lower in OTS-athletes, compared to healthy athletes and to sedentary;
7. All other basal hormones were similar between groups;
8. Basal lactate levels were lower in healthy athletes than non-physically active participants, and also lower than levels in OTS-affected athletes;
9. Creatine kinase (CK) was exacerbated in affected athletes, compared to healthy ones, after similar period since last training with similar training patterns;
10. Neutrophils were higher in healthy athletes than OTS, while lymphocytes were lower compared to sedentary. The neutronphil-to-lymphocyte ratio, a proposed marker of diseases prognosis, was increased in healthy, but not in affected athletes;
11. Catecholamines and the catecholamine-to-metanephrine ratio were exacerbated in OTS, compared to healthy athletes;
12. Healthy athletes had benefits from training in terms of vigor, fatigue, irritability, humor, tension, and lucidity moods, when compared to non-active participants, which were lost in OTS sedentary;
13. Healthy athletes had higher measured-to-expected basal metabolic rate (BMR) ratio and fat oxidation than sedentary and OTS;
14. Healthy athletes had lower body fat, higher muscle mass, and were better hydrated than OTS-affected athletes and sedentary.

These findings, including a total of 50 novel markers and processes identified in both healthy and OTS-affected athletes, supported the hypothesis of the existence of multiple adaptations of clinical, metabolic, biochemical, and body parameters that athletes, while the majority of the physiological adaptive changes are compromised in OTS, which may explain the hallmark of OTS, the loss of performance.

Associations, interactions, synergisms, stimulations, predictions, and inhibitions were further evaluated in joint *post-hoc* analyses of the primary findings of the EROS study, using different and more complex statistical analyses (e.g., multivariate linear regression, logistic regression, and linear correlation analyses).

In terms of biochemical parameters as correlated with other behaviors performed in the EROS-CORRELATIONS (13), further findings were identified:

1. Testosterone: estradiol T:E ratio predicted measured-to-predicted basal metabolic rate (BMR) ratio;
2. T:E ratio and total testosterone level were inversely predicted by fat mass;
3. Estradiol was not predicted by any clinical or biochemical parameter;
4. GH, cortisol, and prolactin responses to an ITSS were strongly correlated between them;
5. Hormonal responses to the ITT were positively correlated with fat oxidation, predicted-to-measured BMR ratio, muscle mass, and vigor, and inversely correlated with fat mass and fatigue;
6. Salivary cortisol 30 min after awakening and the T:E ratio were inversely correlated with fatigue;
7. Tension was inversely correlated with libido and directly correlated with body fat;
8. Predicted-to-measured BMR ratio was correlated with muscle mass and body water;
9. Fat oxidation was directly correlated with muscle mass and inversely correlated with fat mass;
10. Muscle mass was directly correlated with body water;
11. Extracellular water was directly correlated with body fat and inversely correlated with body water and muscle mass.

In summary, overall hypothalamic-pituitary responses to stimulation were diffuse and indistinguishable between the different axes, late hormonal responses, cortisol after awakening and T:E ratio were correlated with vigor and fatigue, T:E ratio was correlated with body metabolism and composition, testosterone was predicted by fat mass, and estradiol predicted anger. Hydration status was inversely correlated with edema, and inter-correlations were found among fat oxidation, hydration, and body fat.

In regards with the most important modifiable habits, also termed as “modifiable patterns,” and which include eating, training, sleeping, professional, and social characteristics, the EROS-DISRUPTORS arm (14) demonstrated among OTS-affected athletes that three dietary patterns, including daily carbohydrate, daily protein, and daily overall calorie intake, were found to be, each one alone, independent triggers of OTS. Conversely sleeping, social, and training patterns depended on the combination with other factors to induce OTS. This arm also demonstrated that once triggered, OTS was inherently able to induce further reductions of cortisol, GH, and adrenocorticotropic hormone (ACTH) responses to stimulations, T:E ratio, neutrophils, neutrophil-to-lymphocyte ratio, vigor levels, hydration status, and muscle mass, while increase of tension levels and visceral fat, independently of other factors.

Despite the novel findings in the healthy and OTS-affected athletes and the learnings from the EROS-CORRELATIONS and EROS-DISRUPTORS arms, we were unable to identify how the modifiable habits can predict or modulate the behavior of basal and stimulated hormonal levels, biochemical, muscular, inflammatory, and immunologic levels, and psychological, and physical metabolism and composition parameters in athletes, when irrespective of OTS.

We hypothesized that a balance between training, resting, and nutrition is crucial for the occurrence of the multiple beneficial adaptations that have been detected in athletes. Hence, in the present study, named as EROS-PREDICTORS, we aimed to identify the influence of each habit patterns evaluated in the EROS study (eating, social, and sleep patterns) on the behaviors of the clinical, metabolic, and hormonal parameters, and when and how these patterns can dysfunctionally modify these behaviors, leading to OTS.

Remarkably, unlike EROS-DISRUPTORS, in which modifiable behaviors were evaluated as potential triggers for OTS, the present manuscript analyzes how modifiable habits shape the clinical and biochemical behaviors, irrespective of the presence of OTS. The sample analyzed in the EROS-DISRUPTORS were those affected by OTS vs. healthy athletes, whereas in this manuscript athletes were analyzed altogether, considering the fact that OTS is a result of a *continuum* process (4, 5) of the physiological adaptations in athletes.

## Materials and Methods

### Subject' and Parameters' Selection

The full participant selection process and primary results of the EROS study were previously presented (6–9). The raw data can be accessed at https://osf.io/bhpq9/. This study was approved by the ethical committee of the Federal University of São Paulo (approval number: 1093965). All subjects gave written informed consent in accordance with the Declaration of Helsinki.

Participants were recruited through sports coaches and social media. Age, sex, weight, and height, and intended to participate in (if suspected for Overtraining Syndrome: OTS; if healthy athlete: ATL; and if non-physically active: NPAC) were questioned prior to a first face-to-face interview.

Exclusion criteria included: extremes of age (<18 y/o and >50 y/o), undertrained athletes (training <300 min/week, <moderate-to-intense intensity, and <6 months consecutively), misdiagnosis of OTS (lack of unexplained decreased performance, presence of confounding dysfunctions that could be the cause of decreased performance), use of drugs or hormones, and altered biochemical or hormonal levels, that may also justify the reduced performance (6–12).

In the present study, from the 117 parameters evaluated by the EROS study (6–9), we elected those were not qualitative, intrinsically linked to other parameters, unvalidated, or missed in more than 5% of the participants, in a total of 76 parameters, from two groups of athletes (OTS-affected and healthy athletes; 39 participants) of the four arms of the EROS study (Table 1) (6–9). From the elected parameters, we excluded those that were not influenced by modifiable patterns, as they were unaltered between the groups of athletes, irrespective of the modifiable patterns.

**Table 1:** Markers evaluated by the EROS study and included in the present analysis.

**Study/Tests (76 parameters)**	**Markers**
**EROS-HPA axis−15 parameters**	
Basal ACTH and cortisol and their response to an insulin tolerance test (ITT)	(1) Basal ACTH (pg/mL), and (2) cortisol (μg/dL) (3) ACTH, and (4) cortisol during hypoglycemia (5) ACTH, and (6) cortisol 30 min after hypoglycemia (7) ACTH, and (8) cortisol increase during ITT
Cortisol response to a *cosyntropin* stimulation test (CST)	(9) Cortisol at 30 min, and (10) at 60 min after injection
Salivary cortisol rhythm (SCR)	(11) Salivary cortisol (ng/dL) at awakening, and (12) 30 min after (13) at 4 p.m. and (14) at 11 p.m. (15) Cortisol awakening response (CAR)
**EROS-STRESS−11 parameters**	
GH and Prolactin response to an ITT	(1) Basal (GH) (μg/L), and (2) prolactin (ng/mL) (3) GH, and (4) prolactin during hypoglycemia (5) GH, and (6) prolactin 30 min after hypoglycemia (7) Prolactin increase during ITT
Glucose behavior during an ITT	(8) Basal serum glucose (mg/dL) (9) Serum glucose during hypoglycemia (mg/dL) (10) Adrenergic symptoms during hypoglycemia (0–10) (11) Neuroglycopenic symptoms during hypoglycemia (0–10)
**EROS-BASAL−26 parameters**	
Hormonal markers	(1) Total testosterone (ng/dL), and (2) Estradiol (pg/mL) (3) IGF-1 (pg/mL), (4) TSH (μUI/mL), and (5) Free T3 (pg/mL) (6) Total catecholamines, and (7) metanephrines (both μg/12 h) (8) Noradrenaline, (9) Epinephrine, and (10) Dopamine (all μg/12 h) (11) Metanephrines, and (12) Normetanephrines (both μg/12 h)
Biochemical markers	(13) Erythrocyte sedimentation rate (ESR, mm/h), and (14) Hematocrit (%) (15) C-reactive protein (CRP, mg/dL), and (16) Lactate (nMol/L) (17) Vitamin B12 (pg/mL), and (18) Ferritin (ng/mL) (19) Neutrophils, (20) Lymphocyte, and (21) Eosinophils (all /mm^3^) (22) Creatine kinase (CK, U/L)
Ratios	(23) Testosterone-to-estradiol, and (24) Testosterone-to-cortisol ratios (25) Neutrophil-to-lymphocyte, and (26) Platelet-to-lymphocyte ratios
**EROS-PROFILE−24 parameters**	
General patterns	(1) Duration of night sleep (h), and (2) Self-reported sleep quality (0–10) (3) Self-reported libido (0–10) (4) Number of hours of activities besides professional training (h/day)
Eating patterns	(5) Calorie intake (kcal/kg/day) (6) Carbohydrate intake (g/kg/day) (7) Protein intake (g/kg/day) (8) Fat intake (g/kg/day)
Psychological patterns	(9) Profile of Mood State (POMS) questionnaire (total score: −32 to +120) (10) Anger (0–48), and (11) Confusion subscales (0–28) (12) Depression (0–60), and (13) Vigor subscales (0–32) (14) Fatigue (0–28), and (15) Tension subscales (0–36)
Body metabolism analysis	(16) Measured-to-predicted basal metabolic rate (BMR, %) (17) Percentage of fat burning compared to total BMR (%)
Body composition	(18) Body fat percentage (%), and (19) Muscle mass weight (kg) (20) Body water percentage (BW, %), and (21) Extracellular water compared to total BW (%) (22) Visceral fat (cm^2^), (23) Waist circumference (cm), and (24) chest-to-waist circumference ratio

For the present analysis, from a total of 51 selected participants divided into three groups (OTS = 14; ATL = 25; and NPAC = 12), the two groups of athletes (OTS and ATL groups) were included, in a total of 39 participants. Non-active participants were not included, as we aimed to be identify behavioral predictions in athletes, not sedentary.

For the evaluation of the modifiable habits, we performed a 7-day specific dietary record, which was followed regularly for at least 3 months. Sleeping duration and quality was self-reported, while specific questions regarding social, professional, and cognitive aspects were performed, as specified previously (6–12).

### Statistical Analysis

**Figure 1:**
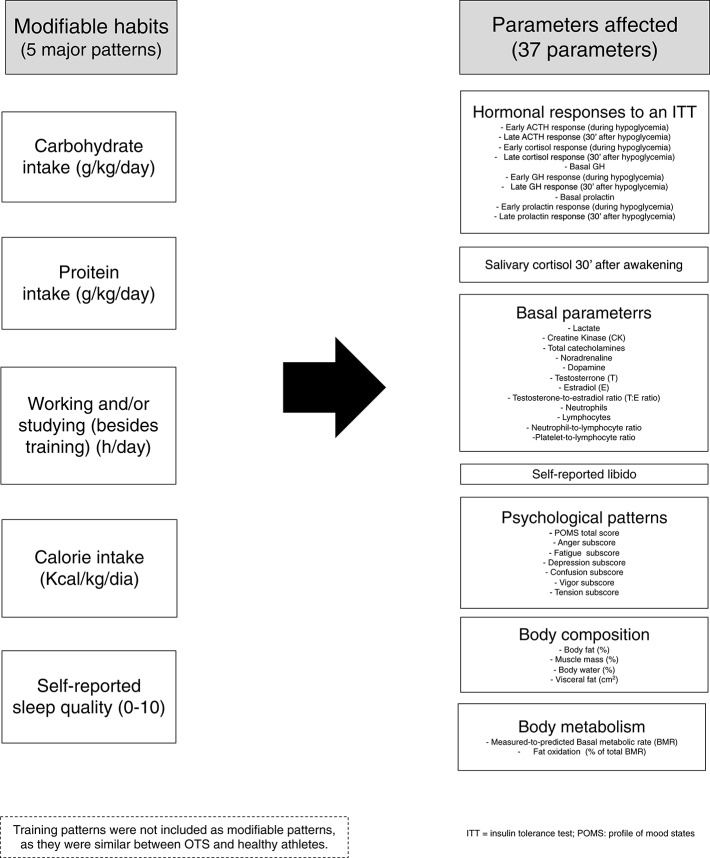
Non-similar modifiable patterns and parameters included in the present analysis.

For the five modifiable patterns (caloric-, carbohydrate-, and protein intake, sleep quality, and the duration of concurrent cognitive activity) and 37 parameters that yielded significant differences between healthy and OTS athletes (Figure 1), in a total of 42 variables, we used multivariate linear regression with the five modifiable patterns as the independent variables and the 37 non-similar clinical and biochemical markers as the dependent variables.

Multivariate linear regression analyzes were performed using the backward method of variable selection method (removal criterion = *p* > 0.01) to analyze the significance of the contributions of the 42 variables, including the five modifiable patterns and 37 non-similar parameters (Figure 1).

The significance of the contribution of the variable to the model was estimated and compared to the removal criteria (*p* > 0.01). When a potential predictor met the removal criteria, it was removed from the regression model. The model was then re-estimated for the remaining variables, and the process was repeatedly performed until none of the predictors achieved the removal criteria. The standardized residual variables of the last model analyzed were examined for normality and homoscedasticity criteria. The cutoff for the presence of multicollinearity was a tolerance index 0.40^3^ for the variables in the last model. A *p* < 0.05 was considered statistically significant for the independent predictors. All statistical analyses were performed using SAS 9.4 (SAS Institute, Inc., Cary, NC).

Given the context of the present study and its main objective, the number of participants in the present study was found to be sufficient for the number of variables and outcomes for both multivariate logistic regression analyses. Compared to previous studies, we consider that we performed a broad and comprehensive analysis, encompassing multiple aspects.

Once this as a complex and multifactorial disorder, we considered that the lack of previous understanding of OTS may have been due to the lack of evaluation of multiple aspects. In addition, it is noteworthy that the level of statistical analysis employed in the present manuscript cannot be found previously in studies on endocrinology of physical activity and sport and on OTS.

In terms of correlations, although *r* > 0.4 (*p* < 0.01) is generally considered to be of moderate association, there is no rule or universally accepted sizes of correlation to be considered as weak, moderate, or strong. Since we studied entirely different biological aspects, and each of these aspects is extensively influenced by a large number of different predictors from different natures, it is unlikely to find a single linear correlation > 0.5 (>−0.5), since each parameter tends to be driven by multiple factors. Hence, in this particular case, according to the literature, a correlation > 0.4 is sufficient to be considered as a strong correlation, or at least moderate-to-strong. The *p*-value for the linear correlations was lower and partial correlations were not considered to avoid incidental misinterpretative correlations.

Parameters that were independently influenced by the presence of OTS were adjusted according to the level of its influence, aiming to homogenize the groups of athletes. These results were published in the EROS-DISRUPTORS arm (14), and included: (1) cortisol 30 min after hypoglycemia, in response to an ITT (26.1% of influence by OTS); (2) cortisol increase during ITT (22.0%); (3) GH 30 min after hypoglycemia, in response to an ITT (23.0%); (4) testosterone-to-estradiol (T:E) ratio (30.7%); (5) neutrophils (13.8%); (6) neutrophil-to-lymphocyte ratio (13.6%) (7) Profile of Mood States (POMS) vigor subscale (83.6%); (8) POMS fatigue subscale (85.7%); (9) POMS tension subscale (42.8%); (10) muscle mass (33.7%); (11) body water (50.5%), and (12) visceral fat (38.2%). Parameters that were not modified by the presence of OTS did not require adjustments according to the population (if OTS-affected or if healthy athletes), since these markers behaved independently from OTS. In addition orrelations that were unlikely to have any biological plausibility were excluded.

Compared to the EROS-DISRUPTORS arm, since this arm had a larger number of variables (total of 44) and demonstrated sufficient statistical power for the present analysis, in the EROS-PREDICTORS, in which we employed a lower number of variables (42 parameters), statistical power was sufficient (6–14). Indeed, largely consistent differences between athletes, strict linear correlations, and small number of outsiders were aspects that strengthen the statistical power of the present study. The raw statistical analysis is also available at the depository (https://osf.io/bhpq9/).

It is important to highlight that the findings in the present are should be considered as suggestive, instead of conclusive, regardless.

## Results

**Figure 2:**
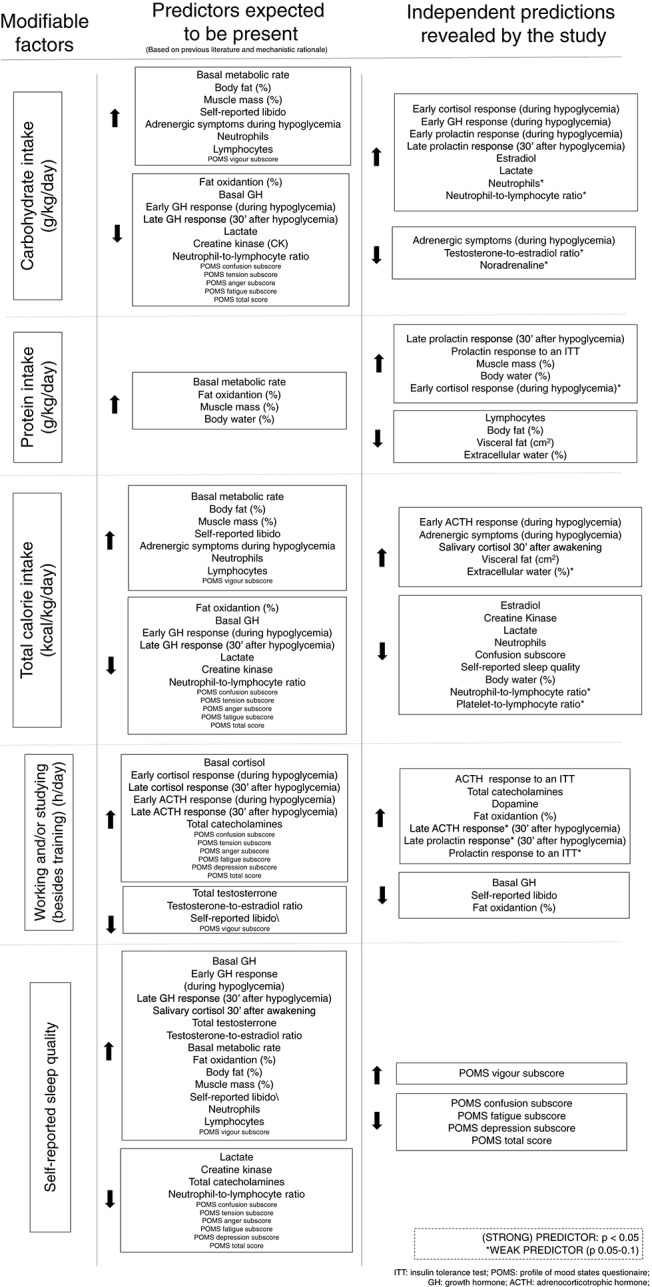
Expected and actual predictions of each modifiable factor.

The results of the multivariate linear regression analyses, including *p*-values, level of association of the independent predictors, and the proposed equations for the estimation of each marker for modifiable factors are detailed in Tables 2–5. A summary of expected (according to biological plausibility for causal relationships and previous scientific data) and actual predictions are shown in Figure 2.

**Table 2:** Modifiable patterns as independent predictors of hormonal responses to stimulations (multivariate linear regression analysis).

**Parameter**	***p* of the influence of the modifiable variables**	**Level of influence by the modifiable variables (Adjusted *R*-Square)**	**Modifiable variables with significant correlations (and *p*-value)**	**Equation for the estimation of the parameter level in male athletes**
**Hormonal responses**				
**to stimulations**				
Early cortisol response to an ITT (during hypoglycemia) (μg/dL)	0.029	23.8%	(1) CHO intake (direct) (*p* = 0.025)	Cortisol (μg/dL) = 8.33 + 0.5 × (CHO intake) + 1.36 × (protein intake)
Late cortisol response (30′ after hypoglycemia) (μg/dL)	0.0005	26.1%	(1) Presence of OTS (inverse) (*p* = 0.0005)	Cortisol (μg/dL) = 17.86 – 3.81 (if OTS)
Early ACTH response to an ITT (during hypoglycemia) (pg/mL)	0.012	17.5%	(1) Calorie intake (direct) (*p* = 0.0035)	ACTH = −67.74 + 2.83 × (calorie intake) + 0.92 × (Total POMS)
Late ACTH response to an ITT (30′ after hypoglycemia) (pg/mL)	0.007	19.9%	(1) Presence of OTS (inverse) (*p* = 0.002)	–
Cortisol response to an ITT (μg/dL)	0.004	22.0%	(1) Presence of OTS (inverse) (*p* = 0.0017)	–
Basal GH (μg/L)	0.033	9.3%	(1) Extra-activities (inverse) (*p* = 0.033)	GH (μg/L) = 0.97 – 0.08 × (extra activities)
Early GH response to an ITT (during hypoglycemia) (μg/L)	0.017	12.0%	(1) CHO intake (direct) (*p* = 0.017)	GH (μg/L) = −0.78 + 1.29 × (CHO intake)
Late GH response (30′ after hypoglycemia) (μg/L)	0.0012	23.0%	(1) Presence of OTS (inverse) (*p* = 0.0012)	–
Early prolactin response to an ITT (during hypoglycemia) (ng/mL)	0.009	15.0%	(1) CHO intake (direct) (*p* = 0.009)	Prolactin (ng/mL) = 8.36 + 2.43 × (CHO intake)
Late prolactin response (30′ after hypoglycemia) (ng/mL)	0.0002	37.8%	(1) Protein intake (direct) (*p* = 0.0004) (2) CHO intake (direct) (*p* = 0.038)	Prolactin (ng/mL) = −28.49 + 1.60 × (CHO intake) + 10.64 × (protein intake) + 2.46 × (extra activities)
Prolactin response to an ITT (ng/mL)	0.0133	17.0%	(1) Protein intake (direct) (*p* = 0.0036)	Prolactin (ng/mL) = −356.25 + 108.6 × (protein intake) + 30.57 × (extra activities)

*CHO, Carbohydrate; ITT, Insulin tolerant test; POMS, Profile of mood states; BMR, Basal metabolic rate; T/E, Testosterone-to-estradiol; OTS, Overtraining syndrome; Calorie intake, kcal/kg/day; CHO intake, g(CHO)/kg/day; protein intake, g(protein)/kg/day; extra activities, working and/or studying hours besides training; sleep quality, self-reported sleep quality (0–10)*.

**Table 3:** Modifiable patterns as independent predictors of basal hormones and biochemical parameters (multivariate linear regression analysis).

**Parameter**	***p* of the influence of the modifiable variables**	**Level of influence by the modifiable variables (Adjusted *R*-Square)**	**Modifiable variables with significant correlations (and *p*-value)**	**Equation for the estimation of the parameter level in male athletes**
**Basal hormones**				
Estradiol (pg/mL)	0.008	20.3%	(1) Calorie intake (inverse) (*p* = 0.002) (2) CHO intake (direct) (*p* = 0.013)	Estradiol (pg/mL) = 50.28 – 0.68 × (calorie intake) + 2.32 × (CHO intake)
Testosterone-to-oestadiol ratio (T/E)	0.0007	30.7%	(1) Presence of OTS (inverse) (*p* = 0.0002)	T/E = 14.1 – 0.86 × (CHO intake) + 12.9 (in case of OTS)
Total nocturnal urinary catecholamines (mg/12 h)	0.0187	11.7%	(1) Extra activities (direct) (*p* = 0.0187)	Total NUC = 49.5 + 20.6 × (extra activities)
Dopamine (mg/12 h)	0.0136	13.1%	(1) Extra activities (direct) (*p* = 0.0136)	Dopamine = 25.7 + 20.1 × (extra activities)
**Basal biochemistry**				
Creatine kinase (CK)	0.02	11.3%	(1) Calorie intake (inverse) (*p* = 0.02)	CK = 1488 – 20.5 × (calorie intake)
Lactate	0.0035	22.9%	(1) Calorie intake (inverse) (*p* = 0.001)	Lactate = 1.62 – 0.02 × (calorie intake)
Neutrophils (/mm^3^)	0.045	13.8%	(1) Calorie intake (inverse) (*p* = 0.044) (2) Presence of OTS (inverse) (*p* = 0.015)	Neutrophils = 4210 – 60.7 × (calorie intake) + 154.4 × (CHO intake) – 1,724 (if OTS)
Lymphocytes (/mm^3^)	0.025	10.8%	(1) Protein intake (inverse) (*p* = 0.025)	Lymphocytes = 2767 – 207 × (protein intake)

*CHO, Carbohydrate; T/E, Testosterone-to-estradiol; OTS, Overtraining syndrome; Calorie intake, kcal/kg/day; CHO intake, g(CHO)/kg/day; protein intake, g(protein)/kg/day; extra activities, working and/or studying hours besides training; sleep quality, self-reported sleep quality (0–10)*.

**Table 4:** Modifiable patterns as independent predictors of moods and feelings (multivariate linear regression analysis).

**Parameter**	***p* of the influence of the modifiable variables**	**Level of influence by the modifiable variables (Adjusted *R*-Square)**	**Modifiable variables with significant correlations (and *p*-value)**	**Equation for the estimation of the parameter level in male athletes**
**Psychology**				
POMS confusion subscale	0.0002	33.7%	(1) Sleep quality (inverse) (*p* = 0.002) (2) Calorie intake (inverse) (*p* = 0.019)	POMS confusion subscale = 15.25 – 0.92 × (sleep quality) – 0.1 × (calorie intake)
POMS depression subscale	0.0001	30.8%	(1) Sleep quality (inverse) (*p* = 0.0001)	POMS depression subscale = 17.22 – 1.66 × (sleep quality)
POMS vigor subscale	<0.0001	83.6%	(1) Sleep quality (direct) (*p* = 0.0002) (2) Presence of OTS (inverse) (*p* < 0.0001)	POMS vigor subscale = 3.7 + 1.15 × (sleep quality) – 11.96 (if OTS)
POMS fatigue subscale	<0.0001	85.7%	(1) Sleep quality (direct) (*p* = 0.0059) (2) Presence of OTS (direct) (*p* < 0.0001)	POMS fatigue subscale = 24.5 – 0.9 × (sleep quality) + 15.3 (if OTS)
POMS tension subscale	<0.0001	42.8%	(1) Presence of OTS (direct) (*p* < 0.0001)	–
Adrenergic symptoms (0–10)	0.003	23.7%	(1) Calorie intake (direct) (*p* = 0.0008) (2) CHO intake (inverse) (*p* = 0.023)	Symptoms = −0.09 + 0.16 × (calorie intake) – 0.45 × (CHO intake)
Libido (0–10)	0.018	11.9%	(1) Extra-activities (inverse) (*p* = 0.018)	Libido = 10.3 – 0.4 × (extra activities)

*CHO, Carbohydrate; POMS, Profile of mood states; OTS, Overtraining syndrome; Calorie intake, kcal/kg/day; CHO intake, g(CHO)/kg/day; protein intake, g(protein)/kg/day; extra activities, working and/or studying hours besides training; sleep quality, self-reported sleep quality (0–10)*.

**Table 5:** Modifiable patterns as independent predictors of body metabolism and composition (multivariate linear regression analysis).

**Parameter**	***p* of the influence of the modifiable variables**	**Level of influence by the modifiable variables (Adjusted *R*-Square)**	**Modifiable variables with significant correlations (and *p*-value)**	**Equation for the estimation of the parameter level in male athletes**
**Body metabolism and composition**				
Fat oxidation (% of total BMR)	<0.0001 (together with body water and T/E ratio)	58.8%	(1) Extra activities (inverse) (*p* = 0.0001)	Fat oxidation = −66.96 + 2.30 × (body water) + 0.51 × (T/E ratio) – 4.99 × (extra activities)
Fat mass (%)	0.0001	31.0%	(1) Protein intake (inverse) (*p* = 0.0001)	Fat mass = 20.35 – 3.1 × (protein intake)
Muscle mass (%)	0.0006	33.7%	(1) Protein intake (direct) (*p* = 0.0135) (2) Presence of OTS (inverse) (*p* = 0.0282)	Muscle mass = 47.84 + 1.42 × (protein intake) – 3.47 (if OTS)
Body water (%)	<0.0001	50.5%	(1) Protein intake (direct) (*p* = 0.0061) (2) Calorie intake (inverse) (*p* = 0.021) (3) Presence of OTS (inverse) (*p* = 0.001)	Body water = 60.75 + 1.69 × (protein intake) – 0.12 × (calorie intake) – 5.77 (if OTS)
Visceral fat (cm^2^)	0.0002	38.2%	(1) Calorie intake (direct) (*p* = 0.0076) (2) Protein intake (inverse) (*p* = 0.023) (3) Presence of OTS (direct) (*p* = 0.0026)	Visceral fat = 47.4 – 11.9 × (protein intake) + 1.3 × (calorie intake) + 45.1 (if OTS)

*CHO, Carbohydrate; BMR, Basal metabolic rate; OTS, Overtraining syndrome; Calorie intake, kcal/kg/day; CHO intake, g(CHO)/kg/day; protein intake, g(protein)/kg/day; extra activities, working and/or studying hours besides training; sleep quality, self-reported sleep quality (0–10)*.

**Figure 3:**
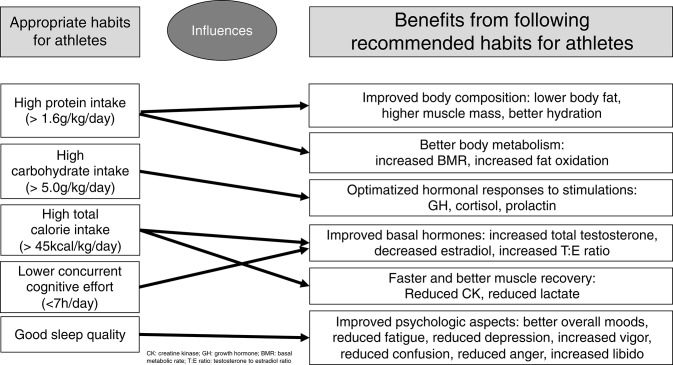
Summary of the influences of modifiable patterns on clinical and biochemical behaviors.

The most significant findings among male athletes regarding eating, sleep, and social patterns as independent predictions are as follows. Carbohydrate intake predicted 12–24% of all early hormonal responses to an ITT, and 37.8% of late prolactin responses when analyzed together with protein intake. Sleep quality and caloric intake inversely predicted 33.7% of the confusion subscale of the POMS questionnaire, and sleep quality predicted vigor and fatigue levels. Protein intake, together with total caloric intake, predicted more than half of the body's water content (within the normal range). Protein intake inversely predicted 31% of the body's fat content; conversely, it independently and positively predicted muscle mass and body water. Caloric intake, but not each macronutrient separately, negatively predicted 10% of creatine kinase (CK) levels, promoting muscle recovery after training sessions, after the training patterns were similar among the athletes. Finally, the amount of working and studying predicted more than 10% of the nocturnal catecholamines, and reduced libido by more than 10%. A summary of the predictions of each modifiable pattern on the behaviors of clinical and biochemical markers, and their consequences, are presented in Figure 3.

## Discussion

The EROS study unveiled adaptations and dysfunctions in acute and chronic hormonal responses to stimulations, other hormones, immunologic, inflammatory, and muscular parameters, and body composition and metabolism in healthy athletes and OTS, respectively, and the correlations between these parameters and eating, social, and sleep patterns. All these findings were conflicting or unclear prior to the present study (15–19).

In the present arm of the EROS study, our objective was to explore and unravel which modifiable factors modulate the clinical, metabolic, and biochemical markers assessed in the EROS study and their mechanisms of action, by employing innovative design and evaluated parameters, *post-hoc* joint analyses were conducted using more complex statistical tools, unlike the techniques used in previous studies with healthy athletes. This helped identify potential independent predictors (independent variables) of evaluated parameters (dependent variables), particularly, when the biological plausibility of the criteria for causality in the relationships were met. The main findings of the EROS study in male athletes are shown in Table 6.

**Table 6:** Most remarkable findings of the EROS study in healthy athletes.

**Study/Tests**	**Remarkable findings in healthy athletes**
	**EROS-HPA axis**
Basal ACTH and cortisol and their response to an insulin tolerance test (ITT)	(1) Prompter cortisol response (compared to non-athletes and OTS-affected athletes) (2) Optimized cortisol response (compared to non-athletes and OTS-affected athletes)
Salivary cortisol rhythm (SCR)	(3) Higher salivary cortisol 30 min after awakening (compared to non-athletes and OTS-affected athletes)
	**EROS-STRESS**
GH response to an ITT	(4) Higher basal GH (compared to non-athletes and OTS-affected athletes) (5) Prompter GH response (compared to non-athletes and OTS-affected athletes) (6) Optimized GH response (compared to non-athletes and OTS-affected athletes)
Prolactin response to an ITT	(7) Prompter prolactin response (compared to non-athletes and OTS-affected athletes) (8) Optimized prolactin response (compared to non-athletes and OTS-affected athletes)
	**EROS-BASAL**
Hormonal markers	(9) Higher total testosterone (ng/dL) (compared to non-athletes and OTS-affected athletes) (10) Higher total catecholamines and noradrenaline (compared to non-athletes)
Biochemical markers	(11) Lower lactate (compared to non-athletes and OTS-affected athletes) (12) Lower neutrophils (compared to non-athletes and OTS-affected athletes) (13) Higher lymphocytes (compared to non-athletes and OTS-affected athletes)
Ratios	(13) Lower neutrophil-to-lymphocyte (compared to non-athletes and OTS-affected athletes)
	**EROS-PROFILE**
General patterns	(14) Better self-reported sleep quality (compared to non-athletes and OTS-affected athletes)
Psychological patterns	(15) Better overall moods, and anger, confusion, vigor, depression, tension, and fatigue subscales (compared to non-athletes and OTS-affected athletes)
Body metabolism analysis	(16) Higher measured-to-predicted basal metabolic rate (BMR) (compared to non-athletes and OTS-affected athletes) (17) Higher percentage of fat burning compared to total BMR (compared to non-athletes and OTS-affected athletes)
Body composition	(18) Lower body fat percentage (compared to non-athletes and OTS-affected athletes) (19) Higher muscle mass weight (compared to non-athletes and OTS-affected athletes) (20) Higher body water percentage (compared to non-athletes and OTS-affected athletes) (21) Extracellular water compared to total BW (compared to non-athletes) (22) Lower visceral fat (compared to non-athletes and OTS-affected athletes)

From the identification of eating, social, and sleep patterns as independent predictors of beneficial or harmful outcomes, we aimed to recommend more precise approaches for the continuous improvement of athletes, by the optimization of eating, social, and sleep habits to improve the performance and the overall well-being of athletes.

Other modifiable factors, such as the use of drugs, hormones, smoking, drinking alcohol, and other social behaviors were exclusion criteria, and therefore, were not analyzed. We intuitively assumed that athletes were fully aware of the need to avoid drugs, anabolic steroids (unless clinically needed), smoking, alcohol intake (except during special social events), and sleep deprivation due to excessive hedonic living.

### Carbohydrate Intake

**Figure 4:**
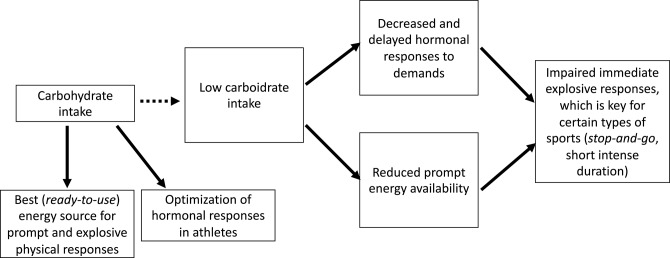
Proposed mechanisms for the impaired performance observed in prolonged low carbohydrate intake.

Carbohydrate intake had multiple effects on the behavior of hormones and other biochemical parameters. It was an independent predictor of the overall early hormone responses to an ITT, accounting for up to 24% of responses (early hormonal responses to an ITT can predict sports performance that demands sudden and explosive reactions). Our hypothesis is that improved responses require a greater availability of energy, and carbohydrates are notorious prompters and an easy source of energy; therefore, which may justify why carbohydrate intake and its consequent prompt availability has been demonstrated to be an independent predictor of early hormone responses to stimulations. Accordingly, we hypothesized that carbohydrate deprivation may have led to decreased and delayed hormonal responses, which would indirectly impair athletes performance, as identified in the primary findings of the EROS study (6, 7) (Figure 4).

In contrast to the suppressive effect of acute carbohydrate intake on GH release (20), chronic carbohydrate intake had a stimulating effect on the GH response, showing a dual effect of carbohydrate intake on GH-release patterns.

Similarly to the dual effects on GH release, carbohydrate intake has also demonstrated an apparent dual effect on aromatase activity (i.e., conversion from testosterone to estradiol) was found. While a very low carbohydrate intake may be related to a pathological increase in aromatase activity (9), which corroborates previous similar findings (21, 22). Notwithstanding, excessive carbohydrate intake may also increase aromatase activity, causing increased estradiol and a decreased testosterone-to-estradiol (T/E) ratio, as observed in our previous findings (9, 15, 16, 18, 19). This finding may justify the not fully elucidated finding of higher estradiol levels in obese males, since higher estradiol levels in these males cannot be not fully explained by the hypertrophy of adipocytes (9). Despite the protective role of overall caloric intake among elite athletes, excessive carbohydrate intake may have a pro-inflammatory role (9, 23), as typical markers of unspecific subclinical metabolic inflammatory states have been correlated with excessive carbohydrate intake, including increased aromatase activity, increased lactate levels without concurrent increase in CK levels (unrelated to muscle stimulation) (9), and slight non-significant increased neutrophils. Neutrophils are independently associated with inflammatory status, and cardiovascular and neoplastic diseases, in the absence of clinical infections or the use of glucocorticoids (24, 25).

Despite claims that lower carbohydrate intake does not impair performance, even for elite athletes (26), higher carbohydrate intake was shown to have positive effects on hormonal profile. Nonetheless, excessive intake has the potential to induce a pathological increase in aromatase activity. In addition, the EROS studies showed carbohydrate intake below 5.0 g/kg/day predicted harmful effects on hormonal responses and performance (6–8, 21–23).

### Protein Intake

Protein intake was found to predict the most important parameters of body metabolism and composition positively, in an independent manner, including increased BMR, fat oxidation, muscle mass, and hydration, while protecting against body and visceral fat, accounting for 30–50% of the variation in body fat. Protein intake significantly and inversely predicted (*p* = 0.029) extracellular water, i.e., it protected against the loss of water from the “third space,” thereby preventing edema. All these findings point to the conclusion that protein is a major determinant of body characteristics (6, 15, 16, 18, 19).

The daily whey protein intake by 88% of the athletes may have contributed to the independent benefits found in the present study, since whey consumption has been independently associated with decreased body fat (27), reduced inflammatory parameters (28), and the prevention of fat weight gain (29).

Overall, higher protein intake for athletes had beneficial effects on metabolism and body composition. Previous caution about protein intake related to concerns about kidney and liver safety has been unsubstantiated (27–29), and the present study reinforces that additional protein intake has several benefits without risks of kidney or liver dysfunctions. The EROS study showed that protein intake should be at least 1.6 g/kg/day (7, 15, 16), which is consistent with the latest sports nutrition guideline for athletes (30) and previous researches (27–29) although there is no evidence for a maximum intake limit.

Indeed, we hypothesized that a higher (“unlimited”) protein intake among male athletes would have a protective role in the body metabolism and composition, without a plateau or inverse effect at any point, at least up to 4.5 g/kg/day.

### Overall Caloric Intake

Overall caloric intake, independent of the macronutrient content, had four major influences: positively predicted salivary cortisol 30 min after awakening, enhanced the speed and quality of muscle recovery, prevented aberrant exacerbations of aromatase activity, and prevented a pathological increase in neutrophils without the presence of an apparent infection.

Higher caloric intake, regardless of its content, may increase elite male athletes alertness in the morning, assumed from the increased salivary cortisol 30 min after awakening, and possibly helps increase the speed of the clearance of markers of muscle recovery (CK and lactate). These findings suggest that unlike the predictions for other outcomes, for muscle recovery higher caloric intake seems to be more important than the amount of each macronutrient.

Despite the positive findings associated with overall caloric intake, this was detected as an independent and direct predictor of visceral but (although not for total fat), and it also was a predictor of lower muscle mass when not accompanied by increase of protein intake. Indeed, carbohydrate abuse is frequently associated with low and insufficient protein intake, leading to sarcopenic obesity (31). Thus, for some aspects of body composition, the source of calories is at least as important as the total caloric intake, once the effect of higher caloric intake when from protein may have opposite effects compared to non-protein higher overall caloric intake.

In conclusion, increase of caloric intake in elite athletes improved the quality of muscle recovery, hormonal environment, and sports performance. The total amount of needed calories was more important than their source. The EROS study found athletes should consume a minimum of 35 kcal/kg/day (6, 9) to achieve this caloric intake. Any macronutrient (i.e., protein, carbohydrate, or fat) can be added to the diet, even if the amount exceeds the athletes daily caloric needs.

### Other Activities

For all elite athletes, excessive concomitant physical and cognitive efforts may lead to harmful effects, although different from those related to insufficient caloric, protein, and carbohydrate intake. The number of hours of studying and/or working was an independent enhancer of ACTH response to stimulation. However, this did not translate into enhanced cortisol release, as would be expected in response to ACTH. The lack of cortisol response to enhanced ACTH release can be hypothesized to be a sort of hypo-responsiveness of the adrenals to ACTH stimulation. Conversely, direct adrenal stimulation in the same participants did not disclose differences in cortisol responsiveness, irrespective of cognitive demands, and was not predicted by any other factor or marker, which weakens this hypothesis.

Basal GH levels were inversely predicted by excessive mental activity, indicating that more studying or working led to lower GH levels when not in pack, although this was not reflected in the GH response to stimulations.

The duration of working and/or studying among elite athletes directly predicted urinary catecholamines. Since catecholamines have acute positive effects on fat oxidation and metabolic rate, a paradoxical reduction in fat oxidation and BMR were detected with increased cognitive activity.

Although catecholamines acutely increase fat oxidation, chronic exposure may have the opposite effect, in a similar manner that happens in hypercortisolism states. Indeed, a chronic *fight-or-flight* readiness effect, typically observed in chronic psychological stress, can lead to fat weight gain and decreased BMR (32), despite the expectedly observed increase of cortisol and catecholamines. Possibly, a decreased sensitivity of the fat tissue to catecholamines is in accordance with a lack of fat loss to be expected under excessive catecholamines (32, 33). The unexpected lack of fat loss has is observed in patients with pheochromocytoma (catecholamine-producing tumors), who are chronically exposed to higher catecholamine levels, or under chronic stress (33).

Considering the present findings and the results from the previous arms of the EROS study, we speculate that when both physical and cognitive demands are concurrently present fat oxidation and BMR get impaired, which is resulted from an environment exposure to chronic stress (6–14, 32). This is correlated with impaired metabolism associated with insufficient resting and recovery, as cognitive stress precludes appropriate physical recovery. Athletes should avoid excessive cognitive activities during periods when volume and intensity of training increase, for example, during seasons. Contrariwise, periods that demand high cognitive effort should not be accompanied by intensification of training load.

### Sleeping

While duration of sleep did not predict any marker or outcome, sleep quality was the most important predictor of psychological outcomes, and the only modifiable factor that modulated overall mood states.

Also, sleep quality was an independent and inverse predictor of total caloric intake; i.e., better sleep quality could be able to reduce overall caloric intake, irrespective of other factors, such as training characteristics. However, greater sleep quality did not lead to additional reduction and consequent insufficient caloric intake.

### Deprivations and Overtraining Syndrome

Collectively, the subjective analysis of the findings of the present study shows that concurrent strict lifestyle in the long run may bring more harms than previously thought. Despite the benefits of adequate caloric and carbohydrate intake, food deprivation, and carbohydrate phobia (“carbphobia”) are present in some athletes, especially those in sports in which categories are based on body weight and body shape is culturally acclaimed, such as high-intensive functional training (HIFT), e.g., CrossFit®, which attempts to simultaneously lower body fat and improve performance (15, 16). These behaviors can lead to fatigue and temporary underperformance, consistent with our finding that lower caloric intake reduces alertness in the morning and impairs muscle recovery, while lower carbohydrate intake may lead to a paradoxical decrease in pace and strength; together these findings are termed overreaching (5). If overreaching is not addressed by an increase in caloric and carbohydrate intake and compensatory rest, athletes can progress to a state of prolonged and hard to recover from decrease in performance, chronic fatigue, and mood disturbances, which characterize classic OTS. In one of the EROS studies, a relatively low caloric (not hypocaloric) and low carbohydrate intake were the two major OTS triggers (6).

Excessive work or studying might lead to multiple harmful effects in athletes, including worsening of hormonal levels, libido, sleep quality, and performance (6, 15, 16, 18, 19). Sleep quality impairs performance, libido, and all psychological functions. We recommend, therefore, against concurrent intense levels of physical and cognitive activity during championships, or intensified training. Athletes should decrease the intensity and duration of studying and/or working, and when more intense studying or working is needed, the volume of training should be decreased. During intensification of training, a maximum work or study duration of 7 h is recommended, following the findings of the EROS study (6).

Multiple modifiable patterns were found to modulate clinical and biochemical behaviors, and we learned answers are unlikely to be found if studies evaluate each aspect separately. The level of importance of each modifiable factor varies by the type of sport. For instance, carbohydrate intake plays an important role in explosive, stop-and-go, and short and intense sports, in which prompter and enhanced hormonal responses and prompter energy availability are the two major factors influencing performance. An overall balance between training, eating, and resting is the most important factor for endurance sports, when prolonged optimization of hormonal responses are desired for a longer time-to-fatigue and a maximum maintenance of pace throughout the training session.

### Limitations

The findings of the EROS study are only applicable for male athletes that practice both endurance and strength exercises, either together (as in high-intensive functional training or CrossFit) or separately (e.g., when athletes practice both weight lifting and middle distance running), as basal and stimulated hormonal and metabolic levels are highly sex-specific and possibly sport-specific. Whether the findings are applicable to exclusive endurance, strength, or explosive sports, is unknown. However, the clinical applications of the present findings can be extrapolated in the absence of more specific data, for practice purposes, as many of the adaptive changes and behaviors found in this study should occur in other populations of athletes. Hence, further studies with larger samples of athletes are crucial to confirm whether our data are reproducible; longitudinal studies are needed because the present study's design precludes drawing conclusions from the sequence of events in response to interventions in modifiable patterns, including training, eating, and social aspects. Additionally, due to unexpected findings regarding changes in hormones and other biochemical markers, for further researches we suggest additional parameters for further studies, including follicle-stimulating hormone (FSH), luteinizing hormone (LH), sex hormone-binding globulin (SHBG), IGF binding globulin-3 (IGFBP-3), tumor necrosis factor-alpha (TNF-alpha), interleukin-1 beta (IL-1beta), CD3, CD4, CD8, CD8/CD4 ratio, lactate dehydrogenase (LDH), free thyroxin (fT4), intra-tissue cortisone:cortisol ratio, and cortisol binding globulin (CBG). Comparisons between exercise-dependent and -independent stimulations should also be performed. Compared to liquid chromatography mass spectrometry/tandem mass (LC/MS-MS/MS), electrochemiluminescence (CLIA) has sufficient relative precision for in-between (pairwise) group comparisons (34–39).

### Final Discussion

The EROS-PREDICTORS arm of the EROS study showed that: (1) carbohydrate intake predicts quick hormonal responses to stress and improves explosive responses during exercise; (2) protein intake improves body composition and metabolism; (3) caloric intake, independent of the its source, predicts muscle recovery; (4) sleep quality improves mood; and (5) excessive concurrent cognitive effort in athletes participating in intense training impairs metabolism and libido. These results support the premise that eating, sleep, and social patterns affect metabolic, hormonal, and clinical behaviors in athletes, and should be addressed to prevent dysfunctions.

## Data Availability Statement

The datasets generated for this study are available on request to the corresponding author and in a depository (https://osf.io/bhpq9/).

## Ethics Statement

The studies involving human participants were reviewed and approved by Ethical Committee of the Federal University of São Paulo (approval number: 1093965). The patients/participants provided their written informed consent to participate in this study.

## Author Contributions

FC and CK developed the central idea of the present manuscript. FC performed the tests of the EROS study, compilated the data, analyzed the results, and participated in the discussions. CK supervised and reviewed the results and actively participated in the discussion. All authors have read and approved the manuscript.

## Conflict of Interest

The authors declare that the research was conducted in the absence of any commercial or financial relationships that could be construed as a potential conflict of interest. The handling editor is currently co-organizing a Research Topic with one of the authors FC and CK, and confirms the absence of any other collaboration.
